# The Cranberry Extract Oximacro^®^ Exerts *in vitro* Virucidal Activity Against Influenza Virus by Interfering With Hemagglutinin

**DOI:** 10.3389/fmicb.2018.01826

**Published:** 2018-08-07

**Authors:** Anna Luganini, Maria E. Terlizzi, Gianluca Catucci, Gianfranco Gilardi, Massimo E. Maffei, Giorgio Gribaudo

**Affiliations:** ^1^Laboratory of Microbiology and Virology, Department of Life Sciences and Systems Biology, University of Turin, Turin, Italy; ^2^Biochemistry Laboratory, Department of Life Sciences and Systems Biology, University of Turin, Turin, Italy; ^3^Plant Physiology Laboratory, Department of Life Sciences and Systems Biology, University of Turin, Turin, Italy

**Keywords:** influenza virus, cranberry extract, Oximacro^®^, dimeric A-type proanthocyanidins, PAC-A2, hemagglutinin, antiviral and virucidal activities

## Abstract

The defense against influenza virus (IV) infections still poses a series of challenges. The current antiviral arsenal against influenza viruses is in fact limited; therefore, the development of new anti-influenza strategies effective against antigenically different viruses is an urgent priority. Bioactive compounds derived from medicinal plants and fruits may provide a natural source of candidates for such broad-spectrum antivirals. In this regard, cranberry (*Vaccinium macrocarpon* Aiton) extracts on the basis of their recognized anti-adhesive activities against bacteria, may provide potential compounds able to prevent viral attachment to target cells. Nevertheless, only few studies have so far investigated the possible use of cranberry extracts as an antiviral tool. This study focuses on the suitability of a cranberry extract as a direct-acting anti-influenza compound. We show that the novel cranberry extract Oximacro^®^ inhibits influenza A and B viruses (IAV, IBV) replication *in vitro* because of its high content of A-type proanthocyanidins (PAC-A) dimers and trimers. Mechanistic studies revealed that Oximacro^®^ prevents attachment and entry of IAV and IBV into target cells and exerts a virucidal activity. Oximacro^®^ was observed to interact with the ectodomain of viral hemagglutinin (HA) glycoprotein, thus suggesting the interference with HA functions and a consequent loss of infectivity of IV particles. Fluorescence spectroscopy revealed a reduction in the intrinsic fluorescence of HA protein after incubation with purified dimeric PAC-A (PAC-A2), thus confirming a direct interaction between HA and Oximacro^®^ PAC-A2. *In silico* docking simulations further supported the *in vitro* results and indicated that among the different components of the Oximacro^®^ chemical profile, PAC-A2 exhibited the best binding propensity with an affinity below 10 nM. The role of PAC-A2 in the anti-IV activity of Oximacro^®^ was eventually confirmed by the observation that it prevented IAV and IVB replication and caused the loss of infectivity of IV particles, thus indicating PAC-A2 as the major active component of Oximacro^®^. As a whole, these results suggest Oximacro^®^ as a potential candidate to create novel antiviral agents of natural origin for the prevention of IV infections.

## Introduction

The influenza viruses type A and type B (IAV, IBV) are widespread major pathogens among human populations and are responsible for seasonal epidemics and pandemics (Paules and Subbarao, 2017). Annual influenza epidemics cause worldwide 3–5 million cases of serious disease and up to half a million deaths among high-risk groups (Molinari et al., 2007; Thompson et al., 2009), with an even greater impact in developing countries (Lafond et al., 2016).

Currently, seasonal vaccines represent the most effective measure to prevent and control IV infections (Houser and Subbarao, 2015). However, prophylaxis and treatment of IV infections may also benefit from two classes of licensed drugs: matrix protein inhibitors and neuraminidase inhibitors (NAIs) (Govorkova and McCullers, 2013; McKimm-Breschkin, 2013; Ison, 2015). Among the first, amantadine and rimantadine inhibit the viral uncoating in endosomes by interfering with the M2 ion channel-mediated acidification (Hay, 1992). However, these drugs are only effective against IAV, and their use is restricted by low effectiveness, collateral effects, and the selection of resistance strains (Hayden and Hay, 1992; Suzuki et al., 2003). On the other hand, oseltamivir and zanamivir are NAIs that prevent the release of IV particles from infected cells, and represent the first-line therapy against influenza infections (McKimm-Breschkin, 2002, 2013; Govorkova, 2013; Ison, 2015). Despite these drugs’ efficacy against both IAV and IBV, the number of circulating NAI-resistant viruses has greatly increased in the past few years (McKimm-Breschkin, 2005, 2013). More recently, peramivir, and laninamivir octanoate have been approved as novel NAIs for the treatment of influenza, and proposed for a combined therapy regimen to prevent resistance occurrence (Ikematsu and Kawai, 2011;Alame et al., 2016). Nevertheless, several strains that are resistant to both of these new NAIs already have been isolated (Takashita et al., 2016). Given these facts, the control of IV infections still presents major challenges, and therefore the development of alternative anti-IV compounds, both effective against antigenically different viruses and characterized by new mechanisms of action, is an urgent priority (Van de Wakker et al., 2017).

Natural products derived from medicinal plants could be exploited as candidates for broad-spectrum antivirals, and polyphenols, flavonoids, glucosides, and alkaloids extracted from several plants and fruits have shown anti-IV activity (Wang et al., 2006; Ge et al., 2010; Ha et al., 2014; Bahramsoltani et al., 2016). Among plant-extracts containing polyphenols, those rich in A-type proanthocyanidins (PAC-A) obtained from the American cranberry *Vaccinium macrocarpon* Aiton (Ericaceae*)* exert antibacterial activities on the basis of their recognized anti-adhesive activity (Gupta et al., 2007; Jepson and Craig, 2008; Shmuely et al., 2012; Blumberg et al., 2013; Krueger et al., 2013). Therefore, cranberry extracts may also represent a source of potential compounds that would prevent viral attachment to target cells as well. Indeed, antiviral activities of cranberry extracts have been reported against Reovirus (Lipson et al., 2007), Enterovirus (Su et al., 2010), and even against IV (Weiss et al., 2005; Oiknine-Djian et al., 2012; Sekizawa et al., 2013). However, a lack of mechanistic studies and the standardization of productive processes to maintain comparable bioactive components concentrations, have limited the development of cranberry products as antivirals against IV infections. More recently, we have observed that the novel cranberry extract Oximacro^®^, which is characterized by a high content of PAC-A, was compelling in the prevention of bacterial infections of the urinary tract (Occhipinti et al., 2016), and was able to exert a potent inhibitory activity against replication of herpes simplex 1 and 2 viruses by inhibiting the attachment of viral particles to target cells (Terlizzi et al., 2016).

Based on these premises, the aim of this study was to evaluate the anti-IV activity of Oximacro^®^. We show that this extract can prevent replication of both IAV and IBV by a mechanism that stems from the inhibition of attachment and entry of IV particles as a consequence of alterations in hemagglutinin (HA) functions. Indeed, dimeric PAC-A were identified as the active component of Oximacro^®^ that interacts with and affects HA. Together, these results indicate that Oximacro^®^ is an attractive candidate to create novel antivirals for the prevention of IV infections.

## Materials and Methods

### Compounds

The cranberry (*V. macrocarpon* Aiton) extract Oximacro^®^ was obtained by Biosfered S.r.l. (Turin, Italy) as a reddish powder, and resuspended in Dulbecco’s Modified Eagle Medium (DMEM; Euroclone) at 10 mg/ml. Purified PAC-A dimer (PAC-A2) was purchased from Extrasynthese (France) and dissolved in 96% v/v ethanol (Sigma-Aldrich, United States) to generate a final concentration of 100 μg/ml. To determine the total PAC content of Oximacro^®^, the Brunswick Labs *p*-dimethylaminocinnamaldehyde (BL-DMAC) assay was performed as previously described by Prior et al. (2010) and Occhipinti et al. (2016). The content of PAC-A and PAC-B of Oximacro^®^ was determined by HPLC-ESI-MS/MS as previously described by Occhipinti et al. (2016). Fractionation of Oximacro^®^ by gel filtration chromatography was performed as described by Terlizzi et al. (2016). Identification of the phytochemical composition of purified fractions (1–5) was carried out by MRM mass spectroscopy (Terlizzi et al., 2016).

Oseltamivir carboxylic acid, the active form of oseltamivir, was obtained from Santa Cruz.

### Cells, Culture Conditions, and Viruses

Madin Darby Canine Kidney cells (MDCK, ATCC^®^ CCL-34^TM^) were propagated in DMEM supplemented with 10% fetal bovine serum (FBS; Euroclone), 2 mM L-glutamine, 1 mM sodium pyruvate, 100 U/ml penicillin, and 100 μg/ml streptomycin sulfate. Infections were performed in the presence of 1 μg/ml of trypsin TPCK treated from bovine pancreas (Sigma-Aldrich, St. Louis, MO, United States) and 0.14% of Bovine Serum Albumin (Sigma-Aldrich, St. Louis, MO, United States).

The influenza A virus strain A/Puerto Rico/8/34 (IAV) and the influenza B virus strain B/Lee/40 (IBV) were a generous gift from Arianna Loregian (University of Padua, Italy). IAV and IBV were cultured and titrated by plaque reduction assay (PRA) on MDCK cells as described below. Experiments were performed under BSL-2 conditions.

### Cytotoxicity Assay

Cytoxicity of either Oximacro^®^ or its purified fractions was determined on MDCK cells after a 72 h of treatment. The number of viable cells was measured by MTT as previously described (Luganini et al., 2008, 2010).

### Antiviral Assays

The antiviral activity of Oximacro^®^, its purified fractions or PAC-A2 was determined by PRA. To this end, MDCK cells were seeded in 24-well plates (3 × 10^5^ cells/well) and after 24 h they were exposed 1 h prior to infection to increasing concentrations of either Oximacro^®^, its fractions or PAC-A2, and then infected with IAV or IBV (40 PFU/well). After virus adsorption (1 h at 37°C), cultures were incubated in medium containing 0.7% Avicel (FMC BioPolymer) plus Oximacro^®^ or its fractions. At 48 h post-infection (h p.i.), the cells were fixed with a solution of 4% formaldehyde in phosphate-buffered saline 1X (PBS) for 1 h at room temperature (RT) and stained with a solution of 1% crystal violet. The microscopic plaques count then allowed to define the concentration of either Oximacro^®^ or its purified fractions that produced 50 and 90% reductions in plaque formation (IC_50_ and IC_90_).

For the time-of-addition assay, a modified PRA was performed. Briefly, MDCK cells were seeded as described above for PRA, and the following day different Oximacro^®^ concentrations were added 1 h before virus adsorption (pre-adsorption treatment), during virus adsorption (adsorption treatment), or after adsorption for 48 h (post-adsorption), as summarized in the scheme depicted in **Figure 2**. The cells were infected with either IAV or IBV (40 PFU/well) for 1 h at 37°C and then washed with warm medium to remove unbound virions. At 48 h p.i., the plaques were stained as described above.

For viral attachment and entry assays, MDCK cells were seeded as for PRA and after 24 h cultures were chilled on ice for 20 min and washed three times with cold medium. For the attachment assay, chilled MDCK cell monolayers were infected at 4°C for 2 h with either IAV or IBV (40 PFU/well) in the presence of different concentrations of Oximacro^®^ (20-10-5-2.5 μg/ml). After viral adsorption, the cells were washed twice with warm medium and incubated with medium-containing 0.7% Avicel for 48 h at 37°C. The IV plaques were then stained as described above.

For the entry assay, MDCK cell monolayers were infected with either IAV or IBV (40 PFU/well) for 2 h at 4°C. After adsorption, infected cultures were washed with cold medium, and different concentrations of Oximacro^®^ (20-10-5-2.5 μg/ml) were added for 2 h at 37°C to allow viral entry. Then, cell monolayers were treated with cold acidic glycine buffer (100 mM glycine, 150 mM NaCl, pH 3) for 30 s to remove adsorbed virions, washed twice with warm medium, and then incubated with medium-containing 0.7% Avicel for 48 h at 37°C. The plaques were then stained as described above.

To evaluate the effect of Oximacro^®^ on IV infectivity, 2 × 10^4^ PFU of either IAV or IBV were incubated with 25 μg of Oximacro^®^ for 0, 60, 120, or 180 min at either 37°C or at 4°C. The, viral suspensions were diluted 100-fold in medium to lower the concentration of Oximacro^®^ below that required for antiviral activity, and residual IV infectivity was measured by PRA as described above.

### Immunoblotting and SDS–PAGE

To investigate the interaction of Oximacro^®^ with IAV Hemagglutinin (HA), aliquots of purified recombinant HA subtype H1N1 (A/Puerto Rico/8/1934) ectodomain (1–528 aa) (Sino Biological-11684V08H50) were incubated at 37°C for different times with Oximacro^®^. Then, mixtures were treated with SDS sample buffer, fractionated through 10% SDS–PAGE, and analyzed either by staining with Coomassie blue or immunoblotting using the rabbit anti-HA antibody (PA5-34929, ThemoScientific). Immunocomplexes were detected with a goat anti-rabbit Ig Ab conjugated to horseradish peroxidase (Life Technologies, Carlsbad, CA, United States) and visualized by enhanced chemiluminescence (Western Blotting Luminol Reagent, Santa Cruz).

### Fluorescence Quenching Assay

PAC-A2 (Extrasynthese) was tested for its ability to bind to HA using fluorescence quenching. In this assay, PAC-A2 quenches the protein’s intrinsic fluorescence produced by the tryptophan fluorophores. Measurements were performed in a quartz cuvette using a fluorescence spectrometer LS55 (PerkinElmer). Fluorescence emission spectra were monitored in the 295–500 nm range using a scan-rate of 200 nm/min upon excitation at 290 nm. The excitation and emission slits were arranged to 8.0 and 10.0 nm, respectively. The protein was dissolved in PBS buffer (pH 7.4) at a concentration of 1.2 μM. Samples were measured in the absence and presence of PAC-A2, following 1 h 25°C. Data were normalized by subtracting the contribution of PAC-A2 fluorescence.

### Ligand and Protein Structure Preparation for Docking Experiments

Ligand and protein structures were prepared using the YASARA structure package (Krieger et al., 2002). YASARA, version 17.5.9, has all the functions embedded useful to predict and validate macromolecular structures, including ligand docking and highly accurate force fields with knowledge-based potentials. Hydrogens were added to both the crystal structure of IAV HA A/Puerto Rico/8/34 (PDB ID: 1RU7; Gamblin et al., 2004) and ligand molecules. All the structures were then optimized through energy minimization using preparing the molecules as previously reported (Catucci et al., 2012; Gao et al., 2016). AMBER 03 force field was employed throughout all simulations. The cutoff for van der Waals interactions was 7.86 Å and the Particle Mesh Ewald (PME) algorithm was applied to compute the long range electrostatic interactions.

### *In silico* Docking

In the docking experiment, the substrate is originally outside the simulation cell and is placed inside the cell by exploiting the Autodock Lamarckian Genetic algorithm, resulting in a series of binding modes classified by the binding energy outputs (Gao et al., 2016). Among these binding modes, the complex protein-ligand bearing the highest binding energy, calculated by YASARA as the mechanical energy required for disassembling a whole into separate parts (where positive energies indicate stronger binding and negative energies equate to no binding), was selected and further refined by local docking. After docking the results were sorted by binding energy and corresponding predicted Kd.

The chemical structures of PAC-A dimers (PAC-A2), PAC-B-2, Kuromaninin, Delphinidin 3-glucoside, Rutin, Quercetin, and Isorhamnetin were obtained from Pubchem. Structures were optimized in terms of molecular geometry optimization using YASARA. The ligands were docked to the crystal structure of IAV HA A/Puerto Rico/8/34 (PDB ID: 1RU7; Gamblin et al., 2004) using the AutoDock algorithm (Morris et al., 1998, 2009) embedded in YASARA. To explore all the possible solutions, 999 runs of global docking were performed using a simulation cell package and 100 runs of flexible docking. A simulation cell (75 Å × 75 Å × 75 Å) was built to incorporate the extracellular domain of HA. YASARA’s scoring function generated both binding energies and predicted dissociation constant (kd) values. Distinct complex conformations were classified when they differed by at least 5.0 Å RMSD.

### Statistical Analysis

All results were obtained by experiments performed in duplicate in at least three independent biological replicates. Statistical analysis by the one-way ANOVA test was performed using the PRISM software version 5.0 (GraphPad Software, Inc.).

## Results

### Oximacro^®^ Contains a High Percentage of PAC-A

The total PAC content of Oximacro^®^ was in line with the values declared in the CoA (366.06 mg/g ± 4.96). Furthermore, analysis by HPLC-ESI-MS/MS revealed a high percentage content of total PAC-A (86.72% ± 1.65), of which the majority corresponded to PAC-A dimers, and only a small amount was of PAC-A trimers.

Fractionation of Oximacro^®^ by Sephadex LH-20 chromatography produced five major fractions whose chemical characterization highlighted the presence of different phytochemicals, such as anthocyanins, flavonoids, and PACs. As shown in **Supplementary Table 1**, fraction 1 contained delphinidin, and cyanidin glycosides; fraction 2 was composed of quercetin and isorhamnetin; fractions 3 and 4 were composed most of PAC-A dimers and of small amounts of PAC trimers; fraction 5 did not show detectable compounds. Oximacro^®^ and its fractions 1–5 were then analyzed for anti-IV activity.

**Table 1:** Antiviral activity of Oximacro^®^ fractions against IAV and IBV.

Fraction	CC_50_ (μg/ml)	IC_50_ (μg/ml)	
		**IAV**	**IBV**
		
1	>200	>50	>50
2	>200	>50	>50
3	149.7 ± 3.1	5.02 ± 1.2	6.02 ± 1.2
4	136.4 ± 2.4	3.24 ± 1.4	4.07 ± 1.8
5	>200	>50	>50


*IC_50_ and CC_50_ values are indicated.*

### Oximacro^®^ and Its PACs-A Inhibit IAV and IBV Replication

**FIGURE 1:**
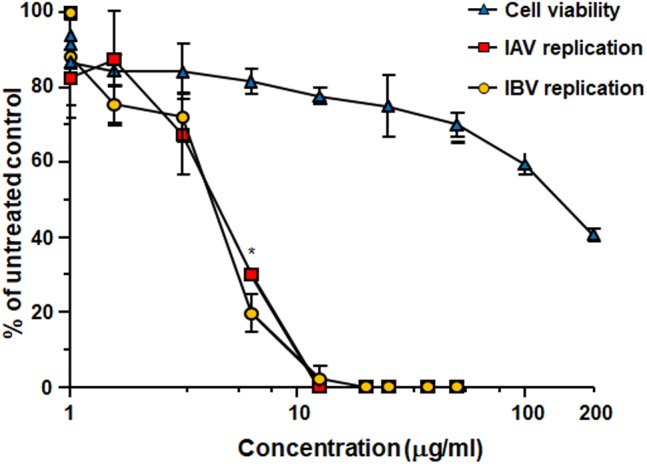
Antiviral activity of Oximacro^®^ against influenza A and B viruses. MDCK cell monolayers were infected with either IAV or IBV (40 PFU/well), and, where indicated, the cells were treated with increasing concentrations of Oximacro^®^ 1 h before, during virus adsorption, and after adsorption throughout the experiment. At 48 h p.i., plaques were stained and microscopically counted. The mean plaque counts for each concentration are expressed as a percentage of the mean plaque count for the control virus. The number of plaques was plotted as a function of Oximacro^®^ concentration; concentrations producing 50 and 90% reductions in plaque formation (IC_50_ and IC_90_) were thus determined. Data represent means ± SD (error bars) of three independent experiments performed in duplicate. Statistical analysis was performed by comparing IAV and IBV replication curves. ^∗∗∗^*p* < 0.001, ^∗∗^*p* < 0.01, and ^∗^*p* < 0.05. To determine cell viability, MDCK cells were exposed to increasing concentrations of Oximacro^®^. After 3 days of incubation, the number of viable cells was determined by the 3-(4,5-imethylthiazol-2-yl)-2,5-diphenyltetrazolium bromide (MTT) method.

Treatment with Oximacro^®^ inhibited both IAV and IBV replication in MDCK cells in a concentration-dependent manner (**Figure 1**). The calculated IC_50_ and IC_90_ values were 4.5 ± 0.2 μg/ml and 10.4 ± 0.2 μg/ml for IAV, and 4.5 ± 0.5 μg/ml and 9.7 ± 0.5 μg/ml for IBV, respectively. The 50% cytotoxic concentration (CC_50_) was 141 ± 0.8 μg/ml, thus indicating that the antiviral activity of Oximacro^®^ did not stem from a non-specific cytotoxicity. The selectivity index for IAV and IBV (CC_50_/IC_50_) was therefore 31.1 and 31.3, respectively.

For comparison, oseltamivir, an approved NAI for the treatment of influenza infections, showed against the tested IV strains, IC_50_ of 0,017 μg/ml for IAV and of 0.22 μg/ml μg/ml for IBV, respectively, and consistent with a previous report (Shin et al., 2017).

To pinpoint the active phytochemical of Oximacro^®^ that was responsible for its inhibitory activity, antiviral assays were performed with purified fractions obtained through fractionation of Oximacro^®^. Analysis of the fractions’ anti-IV activity identified fractions 3 and 4 as having inhibitory activity against IAV and IBV (**Table 1**), thus indicating that the components responsible for the antiviral activity of Oximacro^®^ were PAC-A dimers and trimers.

### Oximacro^®^ Targets Early Phases in the IV Replication Cycle

**FIGURE 2:**
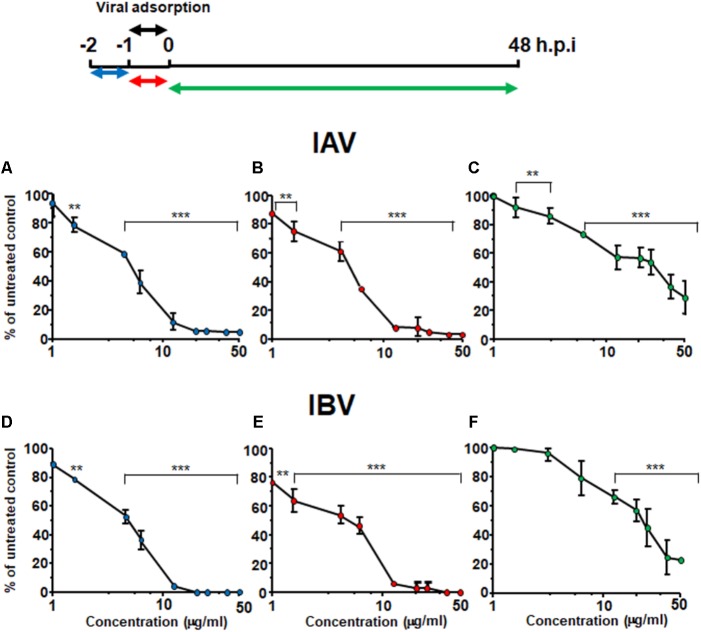
Oximacro^®^ acts in an early stage of IV-replicative cycle. A schematic summary of the time-of-addition experiment is shown at the top of the figure. Accordingly, MDCK cell monolayers were left untreated or treated with different concentrations of Oximacro^®^ from -2 to -1 h (pre-adsorption) **(A,D)** or from -1 to 0 h (adsorption) **(B,E)** before infection with IAV or IBV (40 PFU/well). In the post-adsorption treatment (from 0 to 48 h p.i.) **(C,F)**, untreated and infected cells were exposed to the same concentrations of Oximacro^®^ as indicated in the scheme on the top and were infected with IAV or IBV (40 PFU/well) at -1 h p.i. At 48 h p.i., plaques were stained and microscopically counted. Data represent means ± SD (error bars) of three independent experiments performed in duplicate. For each experimental condition, statistical analysis was carried out by comparing treated samples with the untreated control (100% infection). ^∗∗∗^*p* < 0.001, ^∗∗^*p* < 0.01, and ^∗^*p* < 0.05.

**FIGURE 3:**
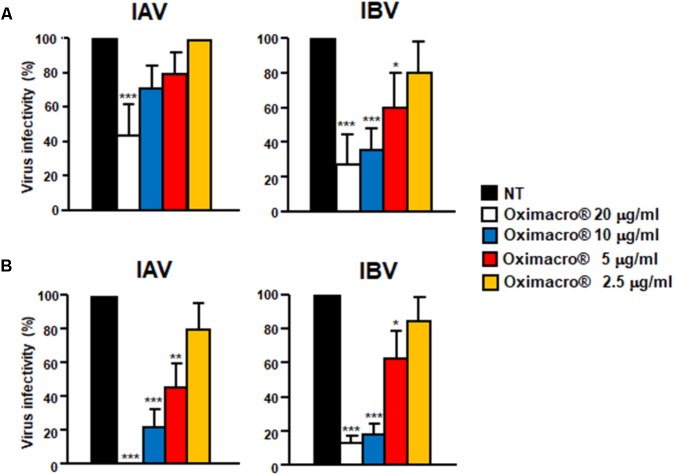
IV attachment and entry is prevented by Oximacro^®^. **(A)** Oximacro^®^ affects IV attachment. Prechilled MDCK cells were infected with precooled IAV or IBV (40 PFU/well) in the presence of different concentrations of Oximacro^®^ (20-10-5-2.5 g/ml) at 4°C for 2 h. After viral adsorption, the cells were washed and overlaid with 0.7% Avicel. At 48 h p.i., viral plaques were stained and microscopically counted. The results shown are means ± SD (error bars) from three independent experiments performed in duplicate. **(B)** Oximacro^®^ inhibits IV entry. Prechilled MDCK cells were infected with IAV or IBV (40 PFU/well) for 2 h at 4°C to allow virion attachment to the cells. After adsorption, the cells were treated with different concentrations of Oximacro^®^ (20-10-5-2.5 μg/ml) for 2 h at 37°C, prior to inactivation of extracellular virus with acidic glycine buffer for 30 s at RT. After further washing, the cells were incubated with medium-containing 0.7% Avicel. At 48 h p.i., viral plaques were stained and microscopically counted. The results shown are means ± SD (error bars) of three independent experiments performed in duplicate. ^∗∗∗^*p* < 0.001, ^∗∗^*p* < 0.01, and ^∗^*p* < 0.05 compared with the 100% virus infectivity of untreated controls.

To understand which phase of the IV replicative cycle is targeted by Oximacro^®^, time-of-addition assays were carried out in accordance with the scheme depicted in **Figure 2**. Briefly, Oximacro^®^ was added: prior to IV adsorption (pre-adsorption stage, from -2 to -1 h prior to infection); during infection (adsorption stage, from -1 to 0 h); or after viral adsorption (from 0 to 48 h p.i.). Evaluation of the IC_50_ indicated that Oximacro^®^ was more effective if added either in the pre-adsorption (IC_50_ of 4.7 μg/ml for IAV and 4.2 μg/ml for IVB, respectively) or adsorption phases (IC_50_ of 4.4 μg/ml for IAV and 5.0 μg/ml for IBV, respectively) than after infection. In fact, addition of the extract at the end of the adsorption period increased the IC_50_ to more than 20 μg/ml for both IAV and IBV, thus indicating that Oximacro^®^ was more active against the early stages of IV replication cycle, such as attachment and entry. To investigate this possibility further, selective attachment and entry assays were performed. In the attachment assays, prechilled MDCK cells were infected with either IAV or IBV in the presence of different concentrations of Oximacro^®^ for 2 h at 4°C, so that to enable the attachment of IV particles, but not their entry into target cells. Oximacro^®^ and unattached virions were then washed away, and the remaining IV infectivity was evaluated at 48 h p.i. As shown, in **Figure 3A**, Oximacro^®^ inhibited the attachment of both IAV and IBV in a concentration-dependent manner. Next, to investigate the inhibitory activity of Oximacro^®^ on IV entry, prechilled MDCK monolayers were infected with IV at 4°C for 2 h to enable viral attachment but not entry. Different concentrations of Oximacro^®^ were then added and infected cultures were incubated at 37°C for 2 h to allow entry of adsorbed IV particles. At the end of this incubation period, IV viruses still bound to the cells were inactivated by briefly treating with acidic glycine buffer, and the remaining IV infectivity was evaluated at 48 h p.i. As depicted in **Figure 3B**, Oximacro^®^ prevented the entry of both IAV and IBV in a concentration-dependent manner. Together, these results revealed that Oximacro^®^ is able to interfere with both viral attachment and entry, therefore suggesting its suitability as an early-acting inhibitor of IV replication.

### Oximacro^®^ Has Virucidal Activity Against IV

**FIGURE 4:**
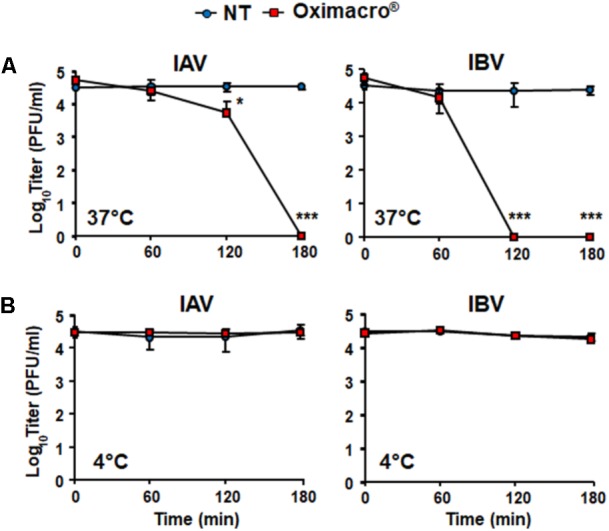
Incubation of IV particles with Oximacro^®^ abrogates infectivity. IAV or IBV (2 × 10^4^ PFU) were incubated at 37°C **(A)** or at 4°C **(B)** for various lengths of time in the absence of Oximacro^®^ (NT) (closed circles) or with 25 g of the Oximacro^®^ (closed squares). After incubation, the samples were diluted to reduce Oximacro^®^ concentration below that which inhibits IV attachment (0.25 μg/ml). Plaques were microscopically counted, and the mean plaque counts was expressed as PFU/ml on a log10 scale. The data shown represent means ± SD (error bars) of three independent experiments performed in duplicate. ^∗∗∗^*p* < 0.001, ^∗∗^*p* < 0.01, and ^∗^*p* < 0.05 compared with the titer of untreated controls (NT).

The observed inhibition of IV attachment and entry could stem from damages caused by Oximacro^®^ to IV particles prior to their interactions with host cells. To verify this hypothesis, suspensions of IAV or IBV particles were incubated with Oximacro^®^ (25 g/ml) for 0, 60, 120, and 180 min at either 37°C or at 4°C. After incubation, mixtures were diluted to reduce the concentration of Oximacro^®^ well below that which prevents virus attachment and entry (**Figure 3**), and then the residual IV infectivity of diluted samples was measured by PRA on MDCK cells. **Figure 4A** shows that the incubation of IV particles with Oximacro^®^ at 37°C, determined a complete loss in infectivity of IVA and IBV after 180 and 120 min of incubation, respectively. In contrast, incubation at 4°C, even at longer times, led to no detectable loss of IV infectivity (**Figure 4B**), thus suggesting that Oximacro^®^ exhibits time-dependent and temperature-dependent inactivation of infectious IV (i.e., virucidal activity). Likewise, a virucidal activity against IAV and IBV was observed for fraction 4, the richest in A type PAC dimers and trimers (data not shown). Thus, these results suggest that the anti-IV activity of Oximacro^®^ stems from its ability to affect IV virions, thus impairing their attachment and entry into host cells.

### Oximacro^®^ Interferes With IV Hemagglutinin

**FIGURE 5:**
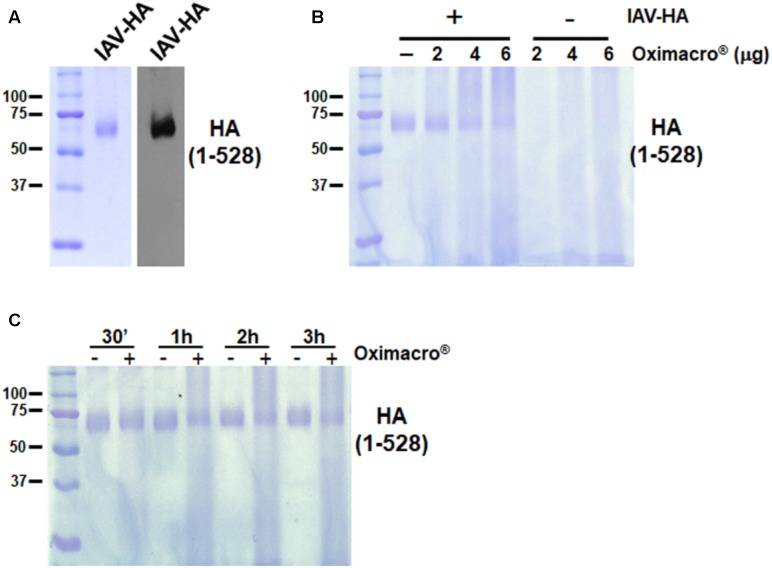
Oximacro^®^ interacts with the recombinant extracellular domain of IAV HA. **(A)** SDS–PAGE and immunoblot analysis of recombinant IAV-HA glycoprotein. Purified HA protein was analyzed on 10% SDS-polyacrylamide gels. Gel was either stained with Coomassie blue or analyzed by immunoblotting with an anti-HA IAV mAb. Left panel: Coomassie blue-stained gel of 1 g of purified recombinant HA-IAV. Right panel: Immunoblot analysis with 200 ng of the same sample as in the left panel. **(B,C)** Oximacro^®^ interacts with recombinant HA in a concentration- **(B)** and time-dependent **(C)** manner. **(B)** Purified recombinant HA-IAV (1 g) was incubated at 37°C with medium or increasing amounts of Oximacro^®^ for 3 h, and then mixtures were analyzed by SDS–PAGE. **(C)** Purified recombinant HA-IAV (1 g) was incubated at 37°C for various lengths of time with medium or 6 g of Oximacro^®^. At 30 min, 1, 2, and 3 h of incubation, mixtures were analyzed by SDS–PAGE. Gels were then stained with Coomassie blue. Sizes are indicated in kilodaltons.

Influenza virus hemagglutinin (HA) is responsible for both the IV attachment and entry by mediating both the initial interaction with cell receptors containing sialic acid, and the fusion between endosome membranes and the viral envelope. Because Oximacro^®^ decreased the infectivity of IV particles (**Figure 4**), we then investigated whether it targets HA, thus impairing its functions in the early stages of the IV replication cycle. To this end, aliquots of purified recombinant HA from the A/Puerto Rico/8/34(H1N1) strain, produced in baculovirus, and corresponding to the ectodomain of the glycoprotein (1–528 aa) (**Figure 5A**), were mixed with increasing concentrations of the extract and incubated at 37°C. **Figure 5B** shows that the exposure of purified HA to Oximacro^®^ caused a reduction in the amount of HA protein band, and altered its electrophoretic mobility with the occurrence of an evident smear toward high molecular weights at the highest Oximacro^®^ concentrations. Similarly, the exposure of HA to Oximacro^®^ for different times determined a time-dependent reduction of the HA protein band, and the appearance of a protein smear with lower electrophoretic mobility compared with the untreated control. Thus, Oximacro^®^ was able to interact with HA in a concentration- and time-dependent fashion. These findings suggest that interactions between Oximacro^®^ and IV HA may hamper its functions in virus attachment and entry, and underlie the overall antiviral activity of Oximacro^®^.

### PAC-A2 Interacts With HA and Exerts a Virucidal Activity

**FIGURE 6:**
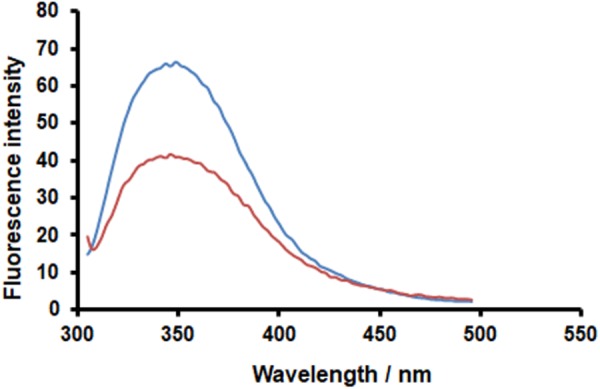
PAC-A2 determines quenching of HA-IAV fluorescence. Recombinant HA-IAV was resuspended in PBS buffer (pH 7.4) at a concentration of 1.2 μM. Fluorescence of HA-IAV aliquots were measured in the absence (blue curve) and presence (orange curve) of 25 M PAC-A2 after incubation for 1 h at 25°C. Fluorescence emission spectra were monitored in the 295–500 nm range using a scan-rate of 200 nm/min upon excitation at 290 nm. Data reported were normalized by subtracting the PAC-A2 fluorescence contribution.

Fluorescence spectroscopy is widely used to investigate enzyme–ligand interactions, because alteration of the fluorescence emission spectrum of proteins can be used to detect conformational changes, binding to ligands, or unfolding processes. In this regard, it has been reported that PACs can reduce the signature fluorescence of bound proteins, thus fluorescence quenching can be used to demonstrate the binding of PACs to proteins (Soares et al., 2009; Gonçalves et al., 2010a,b). Here, we applied fluorescence spectroscopy to test our hypothesis that the binding of Oximacro^®^ to HA is mediated by its PAC-A dimers. The recombinant extracellular domain of our test H1N1 HA (A/Puerto Rico/8/1934) contains nine tryptophans, and the emission maximum of its fluorescence spectrum occurs at 342 nm (**Figure 6**). The addition of 25 μM of purified PAC-A dimers (PAC-A2) caused a reduction in the intrinsic tryptophan fluorescence of HA, but overall it did not markedly modify the shape of the emission spectrum (**Figure 6**). Thus, this result sustains the hypothesis that PAC-A2 binds to HA; moreover, since there was no considerable change in the emission maximum of the intrinsic protein fluorescence, it implies that the interaction does not bring about significant conformational change in the overall structure of HA.

**FIGURE 7:**
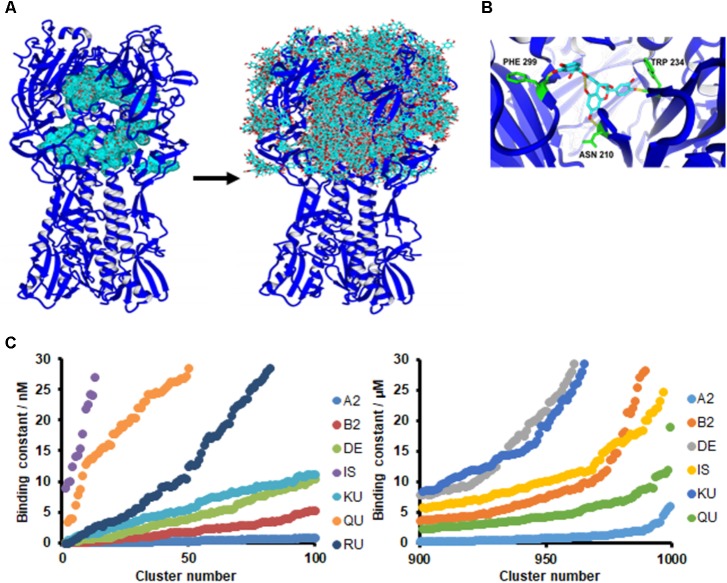
PAC-A2 interacts with HA in an *in silico* model. **(A)** Binding of PAC_A2_ to HA-IAV. Crystal structures of HA-IAV (A/Puerto Rico/8/34-PDB ID: 1RU7) and PAC-A2 were docked using the AutoDock algorithm, embedded in YASARA. Docking analysis revealed that PAC-A2 binds within the internal grooves of the HA structure first (left panel) and subsequently to the surface of the structure (righ panel). **(B)** Best pose of PAC-A2 to HA-IAV. This represents the best complex obtained after 999 docking runs. The HA-IAV structure is represented in blue; PAC-A2 is in cyan; residues forming hydrogen bonds to the ligand are in green; and hydrogen bonds are in yellow. **(C)** PAC-A2 is the Oximacro^®^ component that binds best to HA. Chemical structures of different components of Oximacro^®^ were subjected to docking analysis. PAC-A2 (A2), PAC-B2 (B2), Delphinidin 3-glucoside (DE), Isorhamnetin (IS) Kuromanin (KU), Quercetin (QU), and Rutin (obtained from Pubchem) were docked with HA. In the left panel, the distribution of the binding constants of the top 100 scoring complex clusters is shown; in the right panel, the distribution of the worst 100 scoring complexes is shown. In the right panel, Rutin is not shown because its binding constant was >30 μM.

To support the occurrence of interactions between HA and PAC-A2 further, *in silico* docking simulations were performed. Since PAC are known to bind protein structures at multiple binding sites (Hagerman and Butler, 1981), all the possible solutions were included in the analysis to provide a possible mechanism of interaction between the small PAC-A2 dimer molecule and the HA structure. Using the crystal structure of HA (A/Puerto Rico/8/1934, PDB ID: 1RU7) of our tested IAV strain, docking analysis revealed that PAC-A dimers binds to the internal grooves of the HA structure first (**Figure 7A**, left panel), and subsequently to the surface of the HA structure (**Figure 7A**, right panel). The best binding pose is shown in **Figure 7B**, where HA residues (PHE 299, TRP 234, and ASN 210) forming hydrogen bonds to PAC-A2 are highlighted.

We then performed further *in silico* docking analysis tests to investigate the ability of the different components present in Oximacro^®^ fractions (**Supplementary Table 1**) to interact *in silico* with the crystal structure of HA. Of the different Oximacro^®^ components tested, the PAC-A dimer was identified as the molecule with the best docking capability. In fact, when comparing the theoretical dissociation constants for the top 100 scoring poses (**Figure 7C**, left panel), clustered to separate complexes with a RMSD higher than 5.0 Å, the simulated PAC-A2-HA complexes formed most favorably with an affinity under 10 nM. Furthermore, the distribution of all the docking data (**Figure 7C**, right panel) confirms that even the worst 100 theoretical complexes (out of 999) are still much more likely to bind HA efficiently than any of the other Oximacro^®^ components.

These *in silico* docking simulations are therefore consistent with the fluorescence analysis (**Figure 6**) and support the hypothesis that PAC-A2 constitute the chemical component of Oximacro^®^ responsible of binding to and altering HA.

**FIGURE 8:**
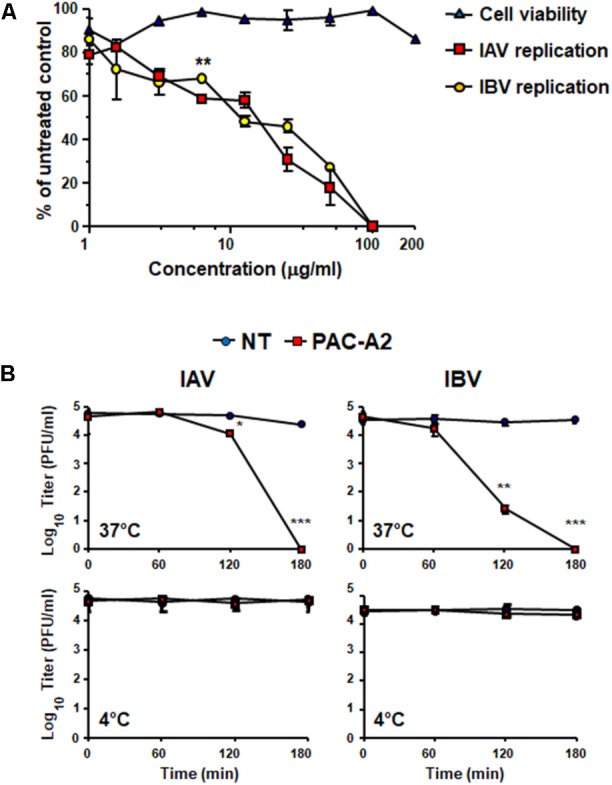
Purified PAC-A2 inhibits IV replication by causing loss of virion infectivity. **(A)** PAC-A2 inhibits replication of IAV and IBV. MDCK cell were infected with either IAV or IBV (40 PFU/well), and, where indicated, the cells were treated with increasing concentrations of PAC-A2 1 h before, during virus adsorption, and after adsorption throughout the experiment. At 48 h p.i., plaques were stained and microscopically counted. To determine cell viability, MDCK cells were exposed to increasing concentrations of PAC-A2, and the number of viable cells was determined by the MTT method. Statistical analysis was accomplished by comparing IAV and IBV replication curves. ^∗∗∗^*p* < 0.001, ^∗∗^*p* < 0.01, and ^∗^*p* < 0.05. **(B)** Treatment with PAC-A2 abolishes the infectivity of IV particles. IAV or IBV (2 × 10^4^ PFU) were incubated at 37°C **(A)** or at 4°C **(B)** for various lengths of time in the absence (closed circles) or in the presence of 50 μg of PAC-A2 (closed squares). After incubation, the samples were diluted to reduce PAC-A2 below that which inhibits IV replication and the residual infectivity assessed by plaque assay. The mean plaque counts were expressed as PFU/ml on a log10 scale. The data shown represent means ± SD (error bars) of three independent experiments performed in duplicate. ^∗∗∗^*p* < 0.001, ^∗∗^*p* < 0.01, and ^∗^*p* < 0.05 compared with the titer of untreated controls (NT).

To verify experimentally the role of PAC-A dimers as antiviral molecules, we tested purified PAC-A2 in some antiviral assays against IV. As shown in **Figure 8A**, PAC-A2 produced a concentration-dependent inhibition of both IAV and IBV. However, its IC_50_ against both IAV and IBV was higher than that of Oximacro^®^: 14.8 μg/ml for IAV, and 11.2 μg/ml for IBV, respectively. This result may suggest the occurrence of a possible synergistic effect of PAC-A2 with PAC-A trimers, since the IC_50_ of fraction 4, the richest in PAC-A dimers and trimers was similar to that of Oximacro^®^ (**Table 1**). Moreover, when tested on IV particles’ infectivity (**Figure 8B**), PAC-A2 determined a complete loss of IAV and IBV infectivity after an incubation of IAV and 180 min at 37°C, which indicated the ability of PAC-A2 to bind to IV particles and thereby impair their infectivity prior to attachment to host cells. Altogether, these results suggest PAC-A dimers as the active components of Oximacro^®^ responsible of the majority of the overall anti-IV activity of the extract.

## Discussion

New anti-influenza molecules need to be continually developed, and this can be done through the adoption of a variety of approaches, such as: (a) improvement of existing drugs; (b) selection and validation of new viral molecular targets; (c) the search for molecule able to inhibit early events in the replication cycle and thus to prevent the establishment of infections; (d) the search for agents able to modulate the host antiviral defense responses; and (e) exploitation of the synergistic effects of compound mixtures acting on different molecular targets (Loregian et al., 2014). Regardless of the approach adopted, a critical prerequisite for developing anti-IV compounds is the requirement for effectiveness against antigenically diverse viruses. In this scenario, natural products derived from plant extracts could be a source of candidates for broad-spectrum anti-influenza compounds able to interfere with early phases of the replication cycle of antigenically different IV viruses. This approach, however, is complicated by a number of hurdles that must be overcome; they include: the production of highly active and standardized extracts; identification of the active components responsible for the antiviral activity; and characterization of the mechanism(s) of action, which are often related to a synergistic cooperation among different components.

In this study, we report that Oximacro^®^, a cranberry extract characterized by a remarkable amount of PAC-A, exerts a potent dose-dependent antiviral activity against IAV and IBV by a virucidal mechanism that involves the interaction with viral particles, and the subsequent impairment of the HA-mediated adsorption and entry into host cells.

Many phytochemicals of relatively low molecular weight, such as polyphenols, flavonoids, terpenes, glucosides, and alkaloids extracted from different plants have been shown to be endowed with an antiviral activity against IV (Wang et al., 2006; Song et al., 2014; Grienke et al., 2016). In particular, among the polyphenols-enriched plants extracts active against IV (Droebner et al., 2007; Mori et al., 2008; Gangehei et al., 2010; Yang et al., 2014), some have been proposed as adjuvant components in therapeutic regimens based on licensed anti-influenza drugs (Bahramsoltani et al., 2016). Nevertheless, the mechanism of action of most of these polyphenol-enriched extracts remains to be defined.

In this regard, it has been observed that the *Rumex acetosa* polyphenol-enriched extract exerts an inhibitory activity against IAV replication by impairing the attachment of viral particles to target cells (Derksen et al., 2014). The B-type PAC dimer (procyanidin B2-di-gallate, PAC-B2) was characterized as the primary antiviral compound of the *R. acetosa* extract and shown to cause HA oligomerization; thus, suggesting an association between the loss of influenza A virus infectivity and HA alterations, which is consistent with the predicted ability of PAC-B2 to bind *in silico* to HA (Derksen et al., 2014). Thus, it was proposed that PAC-B2 of the *R. acetosa* extract can inhibit IAV attachment by directly interacting with virions and affecting HA in a manner that prevents the binding of viral particles to cell receptors (Derksen et al., 2014).

Moreover, it has been reported that non-dialyzable material (NDM) from cranberry juice prevents IV infectivity and attachment (Weiss et al., 2005) and interferes with the enzymatic activity of viral neuraminidase (Oiknine-Djian et al., 2012). In line with this observation, it has also been shown that pomegranate-derived polyphenols exert a virucidal activity against IV by interacting with HA and NA (Sundararajana et al., 2010).

Likewise, here, we report that Oximacro^®^ abolishes both IAV and IBV replication through a virucidal mechanism that involves its direct binding to HA, as indicated by the alteration of electrophoretic mobility of the HA ectodomain upon incubation with Oximacro^®^ (**Figure 5**), and that such interaction can be mediated by PAC-A2 present in the extract (**Figure 6**). However, our direct comparison between PAC-A2 and PAC-B2 by *in silico* docking simulations showed a lower binding affinity to HA of the latter (**Figure 7**).

Taken together, these findings support the view that polyphenols derived from different plants are able to bind with a certain degree of propensity to HA and NA on the IV envelope.

The observed capability of PACs contained in extracts of *R. acetosa* (Derksen et al., 2014) or *V. macrocarpon* (this study) to interact with and affect IV HA, could be a consequent to the natural ability of polyphenols to bind and aggregate proteins (Charlton et al., 2002; Ebraihimnejad et al., 2014). Dealing with possible molecular mechanisms behind this PAC’s effect, it has been proposed that different types of chemical linkages, such as hydrogen bonding, electrostatic interactions, and even covalent bonds may contribute to the formation of protein-PAC complexes (Ebraihimnejad et al., 2014). In this regard, we observed that the exposure of HA-Oximacro^®^ complexes to boiling in SDS sample buffer did not abrogate Oximacro^®^-induced alterations of HA’s electrophoretic mobility (**Figure 5**). Thus, it is possible that the incubation of Oximacro^®^ with the ectodomain of HA may result even in covalent bonding between the two partners. Moreover, our docking simulations (**Figure 7B**) predicted the formation of three hydrogen bonds between the PAC-A2 and Phe99, Asn210, and Trp234 of HA, with a computed binding energy of 14.13 kcal/mol and a dissociation constant of 44.04 pM. Together, the formation of both hydrogen and covalent bonds between the reactive quinone groups of PAC-A dimers and trimers (Ebraihimnejad et al., 2014) and HA may eventually lead to extensive crosslinking of the IV glycoprotein, thus resulting in smearing and reduction of intensity of the HA protein band as observed in interactions experiments with the purified HA ectodomain (**Figure 5**). However, it remains to establish whether these HA’s alterations stem from the binding of PAC-A to specific domains of the IV glycoprotein, or whether PAC-A of Oximacro^®^ simply “upholster” HA surfaces (**Figure 7A**) and, in this manner, they lead to the inhibition of HA functions in the initial stages of the IV replication cycle. In this regard, our docking simulations also suggested that PAC-A2 binds more strongly to the central HA groove, as confirmed by the top 100 possible poses (**Figure 7C**, left panel). Based on this observation, a predicted binding mechanism would entail an initial binding of PAC-A2 to the HA groove and then, once all the highest affinity sites have been saturated, the PAC-A2 would bind to other residues on HA surfaces (**Figure 7C**, right panel).

Overall, the described mechanism of action of Oximacro^®^ against IV advocates its potential application in the prevention of influenza infections. It is therefore possible to envisage that a local application of Oximacro^®^ in the upper respiratory tract, administered in the form of either tablets/chewing gums or through inhaling devices, would allow the active PAC-A to inactivate infecting virus, thus preventing the occurrence of an infection. This proposal may be sustained also by the high safety profile that many formulations of dried cranberry extracts have shown in their widespread use to prevent urinary tract infections (UTIs) (Jepson and Craig, 2008; Blumberg et al., 2013). However, the development of Oximacro^®^ as anti-influenza aid asks its evaluation in animal models of IV infections, to determine whether it should be proposed as a novel active ingredient of broad-spectrum anti-IV agents of natural origin suitable to prevent IV infections.

## Author Contributions

AL, MT, GC, MM, and GGri conceived and designed the experiments. AL, MT, and GC performed the experiments. AL, MT, GC, GGil, MM, and GGri analyzed the data. MM and GGri wrote and revised the manuscript. All authors read and approved the final manuscript.

## Conflict of Interest Statement

MM is also currently a fellow of the Biosfered S.r.l. company. No patents are pending and Biosfered supported the study by providing Oximacro^®^ and partly supporting the research. Biosfered did not interfere with the design, analysis, and decision to publish this paper. The remaining authors declare that the research was conducted in the absence of any commercial or financial relationships that could be construed as a potential conflict of interest.
