# Supplementary material for: The Cranberry Extract Oximacro® Exerts in vitro Virucidal Activity Against Influenza Virus by Interfering With Hemagglutinin
Source: Front Microbiol. 2018 Aug 7;9:1826. doi: 10.3389/fmicb.2018.01826 (PMC6090095; doi:10.3389/fmicb.2018.01826)
Supplement: Supplementary file 1 [file Table_1.DOCX]

Supplementary Material

**The Cranberry Extract Oximacro^®^** **Exerts Virucidal Activity Against Influenza Virus by Interfering with Hemagglutinin**

**Anna Luganini, Maria Elena Terlizzi, Gianluca Catucci, Gianfranco Gilardi, Massimo E. Maffei, and Giorgio Gribaudo**

*** Correspondence:** Giorgio Gribaudo, [giorgio.gribaudo@unito.it](mailto:giorgio.gribaudo@unito.it)

**Supplementary Table 1.** Chemical analysis of fractions of Oximacro® obtained by means of Sephadex LH-20 chromatography.

| Fraction | Compound name | [M-H]**^-^** | [M+H]**^+^** | Fragments |
| --- | --- | --- | --- | --- |
|  |  |  |  |  |
| 1 | Chlorogenic acid | - | 355 | 163, 117 |
|  | Delphinidin-3-*O*-glucoside | - | 597 | 303 |
|  | Delphinidin-3-*O*-hexoside | - | 465 | 303 |
|  | Cyanidin-3-*O*-glucoside | - | 581 | 287 |
|  | Quercetin-gluco-rhamnoside | - | 611 | 465, 303 |
|  | Quercetin-gluco-arabinoside | - | 597 | 465, 303 |
|  | Quercetin-gluco-rhamnoside | - | 611 | 465, 303 |
| 2 | Quercetin-hexoside | 463 | - | 301 |
|  | Myricetin | 317 | - | 179, 165, 151 |
|  | Quercetin | 301 | - | 273, 179, 151 |
|  | Isorhamnetin | 315 | - | 300 |
| 3 | Proanthocyanidin A dimer isomer 1 | 575 | - | 539, 449, 411 |
|  | Proanthocyanidin A dimer isomer 2 | 575 | - | 539, 449, 423, 289 |
|  | Proanthocyanidin A dimer isomer 3 | 575 | - | 539, 449, 423, 285 |
|  | Proanthocyanidin A dimer isomer 4 | 575 | - | 539, 449, 411, 289 |
|  | Proanthocyanidin A dimer isomer 5 | 575 | - | 539, 449, 411, 285 |
|  | Proanthocyanidin A dimer isomer 6 | 575 | - | 539, 449, 411, 285 |
|  | Proanthocyanidin A dimer isomer 7 | 575 | - | 539, 449, 411, 285 |
|  | Proanthocyanidin A dimer isomer 8 | 575 | - | 539, 449, 411, 285 |
|  | Proanthocyanidin A dimer isomer 9 | 575 | - | 539, 449, 423, 411, 285 |
|  | Proanthocyanidin A dimer isomer 10 | 575 | - | 539, 449, 423, 285 |
|  | Proanthocyanidin A trimer isomer 1 | 863 | - | 711, 693, 574, 449, 423 |
|  | Proanthocyanidin A trimer isomer 2 | 863 | - | 711, 693, 574, 449, 411 |
|  | Proanthocyanidin A trimer isomer 3 | 863 | - | 711, 693, 574, 449, 411 |
|  | Proanthocyanidin A trimer isomer 4 | 863 | - | 711, 693, 574, 449, 411 |
|  | Proanthocyanidin A trimer isomer 5 | 863 | - | 777, 693, 574, 449, 411 |
| 4 | Proanthocyanidin A dimer isomer 1 | 575 | - | 539, 449, 411 |
|  | Proanthocyanidin A dimer isomer 3 | 575 | - | 539, 449, 423, 285 |
|  | Proanthocyanidin A dimer isomer 4 | 575 | - | 539, 449, 411, 289 |
|  | Proanthocyanidin A dimer isomer 5 | 575 | - | 539, 449, 411, 285 |
|  | Proanthocyanidin A dimer isomer 6 | 575 | - | 539, 449, 411, 285 |
|  | Proanthocyanidin A dimer isomer 8 | 575 | - | 539, 449, 411, 285 |
|  | Proanthocyanidin A trimer isomer 2 | 863 | - | 711, 693, 574, 449, 411 |
|  | Proanthocyanidin A trimer isomer 3 | 863 | - | 711, 693, 574, 449, 411 |
|  | Proanthocyanidin A trimer isomer 4 | 863 | - | 711, 693, 574, 449, 411 |
|  | Proanthocyanidin A trimer isomer 5 | 863 | - | 777, 693, 574, 449, 411 |
